# Hybridized sine cosine algorithm with convolutional neural networks dropout regularization application

**DOI:** 10.1038/s41598-022-09744-2

**Published:** 2022-04-15

**Authors:** Nebojsa Bacanin, Miodrag Zivkovic, Fadi Al-Turjman, K. Venkatachalam, Pavel Trojovský, Ivana Strumberger, Timea Bezdan

**Affiliations:** 1 Singidunum University, Danijelova 32, 11000 Belgrade, Serbia; 2 Artificial Intelligence Engineering Department, Research Center for AI and IoT, AI and Robotics Institute, Near East University, Mersin 10, Turkey; 3 Department of Applied Cybernetics, Faculty of Science, University of Hradec Králové, 50003 Hradec Králové, Czech Republic; 4 Department of Mathematics, Faculty of Science, University of Hradec Králové, 50003 Hradec Králové, Czech Republic

**Keywords:** Computational models, Software

## Abstract

Deep learning has recently been utilized with great success in a large number of diverse application domains, such as visual and face recognition, natural language processing, speech recognition, and handwriting identification. Convolutional neural networks, that belong to the deep learning models, are a subtype of artificial neural networks, which are inspired by the complex structure of the human brain and are often used for image classification tasks. One of the biggest challenges in all deep neural networks is the overfitting issue, which happens when the model performs well on the training data, but fails to make accurate predictions for the new data that is fed into the model. Several regularization methods have been introduced to prevent the overfitting problem. In the research presented in this manuscript, the overfitting challenge was tackled by selecting a proper value for the regularization parameter dropout by utilizing a swarm intelligence approach. Notwithstanding that the swarm algorithms have already been successfully applied to this domain, according to the available literature survey, their potential is still not fully investigated. Finding the optimal value of dropout is a challenging and time-consuming task if it is performed manually. Therefore, this research proposes an automated framework based on the hybridized sine cosine algorithm for tackling this major deep learning issue. The first experiment was conducted over four benchmark datasets: MNIST, CIFAR10, Semeion, and UPS, while the second experiment was performed on the brain tumor magnetic resonance imaging classification task. The obtained experimental results are compared to those generated by several similar approaches. The overall experimental results indicate that the proposed method outperforms other state-of-the-art methods included in the comparative analysis in terms of classification error and accuracy.

## Introduction

Artificial intelligence (AI) has the goal of creating human-level artificial intelligence (HLAI)^1^ and currently, the leading AI subdomain is machine learning, more precisely deep learning. This type of AI is not focused on representing human behavior as the main goal suggests, but rather focuses on providing practically usable results. In this way, deep learning has achieved the levels of human performance regarding specified tasks, however, the HLAI has not yet been achieved^2^. It particularly excels in the tasks of computer vision, natural language processing (NLP), and speech recognition.

For the program of this sort to successfully accomplish tasks such as mentioned, its architecture should imitate the behavior of the human nervous system^3–5^. As an attempt to create such architecture, convolutional neural networks (CNNs) have been created^6^. The CNNs are inspired by the animal visual cortex and consist of several layers, where each layer, except the first input layer, takes the output of the previous one and sends it to the next one. This behavior forms the basics of the CNN model, as the input becomes more filtered with each layer. This provides more detailed outputs after each layer, while reducing the input complexity to an easier form to process, and at the same time without losing any of the critical feature data. The classic example of this process is with the forming of edges on the first layer, and later on respectively with each layer, the corners and sets of edges, parts of objects and sets of corners and contours, and on the final layer recognition of full objects based on the sets of the previous forming parts is achieved.

However, deep learning models, especially deep neural networks, such are CNNs, suffer from some shortcomings. Two of the most important drawbacks of these models are hyperparameters’ tuning and the overfitting issue^7^. For each specific task, a CNN with certain architecture should be generated, because CNN architecture that performs well on every problem does not exist. The architecture is defined with the number and types of layers, the number of neurons in each layer, the loss function learning rate, type of activation function, etc. These components, which are not trainable, are known as hyperparameters’ and it is very challenging to find their proper values for a specific task. This issue is known in the literature as hyperparameters’ tuning^8^.

The major source of the overfitting issue is that during the training, the model’s weight and biases become well adjusted for a limited amount of training data, which makes the model inefficient in making predictions for previously unseen observations (testing data). In other words, the model will not be able to generalize well. This issue can be also viewed from the perspective of bias variance trade-off^9^.

For solving the issue of overfitting several regularization techniques have been proposed^7^. However, one of the most efficient methods, especially for complex structures such are CNNs, is dropout^10^. The basic idea behind the dropout is removing random units (neurons) from the CNNs along with all their input and output connections. The dropout is controlled by the parameter known as the dropout probability ($dp \in [0,1)$) that determines the percentage of units that are discarded from the model.

However, the dropout probability is another CNNs hyperparameter that should be tuned for each particular problem (dataset). One practical way to address this issue is to use an automated framework that will generate optimal or sub-optimal dropout probability value for a given CNN’s structure and the dataset, instead of finding this value manually by performing “trial and error”. A Group of algorithms that can be very efficient in executing such tasks are metaheuristics.

In this manuscript, an automated framework for determining a proper *dp* value for CNN’s dropout layer, based on the swarm intelligence approaches is presented. From the literature examination, it can be noticed that the first framework for dropout regularization by swarm intelligence was proposed by de Rosa et. al. in 2017^7^, where the authors implemented firefly algorithm (FA), cuckoo search (CS), bat algorithm (BA), particle swarm optimization (PSO) for tackling this issue. However, it also can be noticed the lack of swarm intelligence applications for this task and it can be concluded that the swarm intelligence potential in determining optimal (sub-optimal) dropout probability ratio in CNNs was not investigated enough.

The research proposed in this manuscript represents an extension of the investigation shown in^11^, where the proposed swarm intelligence-based framework for dropout regularization was evaluated for only MNIST and CIFAR-10 datasets. In this manuscript, automated dropout regularization CNNs framework, based on the hybridized sine cosine algorithm (SCA) with the well-known firefly algorithm (FA) swarm metaheuristics, was brought forward and it was first validated against MNIST, CIFAR-10, UPS, and Semeion datasets.

In the second experiment, the proposed algorithm has been validated on magnetic resonance images (MRI) classification task. The SCA is a relatively efficient algorithm proposed in 2016 by Mirjalili^12^, and since it in its basic implementation exhibits some deficiencies, an improved, hybrid SCA version was devised for the purpose of this research. Before applying hybrid SCA for dropout regularization, simulations with CEC2019 benchmark instances were conducted and these results are reported as well in the manuscript. Also, to further investigate the performance of other swarm intelligence algorithms for this problem, besides basic SCA and proposed hybrid SCA, recent whale optimization (WOA) swarm algorithm is implemented and tested for this problem as well.

In the very introduction of this manuscript, some terminology ambiguous should be uncovered. According to some sources, the SCA belongs to the group of math-inspired population-based metaheuristics^13^, while some other sources put the SCA in the group of swarm intelligence^14^. In this manuscript, since the hybridization with other swarm algorithm was performed, the SCA is categorized as a swarm intelligence approach.

Taking into consideration basic research assumptions, the motivation of the proposed investigation is to further improve CNN’s classifications performance and to avoid overfitting by establishing better dropout regularization than other methods which results are reported in the literature by utilizing novel SCA metaheuristics. In line with the common practice in computer science when devising and testing the new or modified algorithm, the novel metaheuristics has first been validated on a set of challenging CEC2019 benchmark functions. Afterwards, the proposed algorithm was applied on the problem of dropout probability estimation and tested on four benchmark datasets. Finally, the algorithm has been validated on a practical MRI classification task. Therefore, according to empirical results presented in “CEC2019 benchmark simulations” and “CNN dropout regularization simulations” Sections , the contribution of the proposed research is twofold: classification accuracy of CNN model used in simulations is enhanced and novel state-of-the-art SCA metaheuristics was devised.

Remain sections of the proposed manuscript are structured as follows: “Background and related work” Section gives a brief theoretical background of CNNs and dropout regularization along with relevant literature review from the swarm intelligence domain. The goal of “Proposed method” Section is to provide a basic description of the original SCA and its drawbacks, as well as to introduce insight into the proposed hybrid SCA metaheuristics. “CEC2019 benchmark simulations” Section provides experimental results and comparative analysis of the proposed method for standard CEC2019 benchmarks following with “CNN dropout regularization simulations” Section, where the results obtained from simulations for CNN’s dropout regularization are reported. Finally, “Conclusion” Section concludes this paper and shows directions of potential further research from this area.

## Background and related work

Human beings are not able to process any of information absorbed by labeling, tagging, and putting it into tables. This creates a limitation for the accurate representation of the information obtained in the computational form. It is inefficient and too complex to process for an individual and to translate the obtained information from the photograph into words and in a way that a program can process them. For this reason, the CNN technology has been widely applied for use in visual tasks^15^ and nowadays it is the most commonly utilized deep learning model^6^. The recent advancements employ facial recognition^16–19^, document analysis^20,21^, medical images classification and diagnostics^22–24^, and an important task of analyzing climate change and extreme weather conditions^25,26^ among many other.

The CNN, besides the input layer, consists of three basic types of layers: convolution, pooling, and the fully-connected (dense) layers. The convolution layers filter the data by applying the convolution operation and the features are extracted by the filters of sizes always smaller than input. The most common filter (kernel) sizes are 3 $\times$ 3, 5 $\times$ 5, and 7 $\times$ 7. When kernels are applied to the input, feature maps are generated. Mathematical representation of the convolution operation on the input vector is represented as follows:

1 $$\begin{aligned} z^{[l]}_{i, j, k} = w^{[l]}_{k}x^{[l]}_{i, j} + b^{[l]}_{k} \end{aligned}$$

where $z^{[l]}_{i, j, k}$ stands for the value of the output feature of the *k*-th feature map at location i, j. Representation of the input is given as *x* at the location *i*, *j*, *w* denotes the filters, and bias is *b*.

Following up the convolution operation is the activation given as:

2 $$\begin{aligned} g^{[l]}_{i, j, k} = g(z^{[l]}_{i, j, k}) \end{aligned}$$

where the $g(\cdot )$ is the non-linear function using the output.

Pooling layers can be global or local and the two most applied types are max and average pooling layers. The pooling function is used to reduce the resolution:

3 $$\begin{aligned} y^{[l]}_{i, j, k} = pooling(g^{[l]}_{i, j, k}) \end{aligned}$$

Fully connected layers in CNNs perform the same operations as in classic ANNs. Typical CNN may consist of several dense layers, where the last layer performs classification by using the softmax for multi-class classification and the sigmoid or tanh activation function for binary classification tasks.

Despite the diverse application that CNN technology offers, it is not without shortcomings. As previously noted in “Introduction” Section, CNNs suffer from overfitting and require ways of avoiding such scenarios^10,27^. The most common methods used to address overfitting are^7^: model simplification, early stopping, data augmentation, and regularization. Many regularization techniques have been proposed, e.g. L1^28^ and L2 regularization (weight-decay)^28^ and dropout^10^. To drop a unit from a layer means that it is removed with all of its connections. The neurons to be dropped are selected randomly and temporarily removed from the training process. The absence of these neurons is believed to result in a network with better generalization because it becomes less sensitive to the weight of those neurons. The basis is to exclude randomly selected hidden individuals during the training phase. The goal is to force neighboring neurons to take over the workload from missing neurons which leads to an increase in independent internal representations. This process is only performed ahead of the classification layer upon the last fully-connected layers. The feed-forward operation is performed as the following equation:

4 $$\begin{aligned} z^{[l+1]}_i= & {} w^{[l+1]}_iy^l+b^{[l+1]}_i \end{aligned}$$

5 $$\begin{aligned} y^{[l+1]}_i= & {} g(z^{[l+1]}_i) \end{aligned}$$

where the symbols represent the following terms: *l* is the *l*-th hidden layer of the network, *z* and *y* are input and output vectors respectively, *w* is the weight vector, *b* is bias, and *g* represents the activation function.

The feed-forward operations are used after the dropout regularization as follow^11^:

6 $$\begin{aligned}&r^{[l]}_j\sim Bernoulli(p) \end{aligned}$$

7 $$\begin{aligned}&{\tilde{y}}^{[l]}=r^{[l]} \cdot y^{[l]} \end{aligned}$$

8 $$\begin{aligned}&z^{[l+1]}_i=w^{[l+1]}_i{\tilde{y}}^l+b^{[l+1]}_i \end{aligned}$$

9 $$\begin{aligned}&{\tilde{y}}^{[l+1]}_i=g(z^{[l+1]}_i) \end{aligned}$$

where *r* denotes the vector of independent Bernoulli random variables.

Parameter dropout probability (*dp*), which controls the number of dropped neurons expressed as a percentage, is not trainable and represents CNN’s hyperparameter. Since its value is continuous within the range [0, 1], finding a proper value of this parameter for a specific problem at hand (dataset) is an NP-hard challenge. Finding the proper value of this parameter falls into the category of both CNNs challenges, overfitting avoidance by using dropout regularization and hyperparameter optimization. Metaheuristics, especially nature-inspired ones like swarm intelligence showed as efficient methods for tackling NP-hard problems.

Swarm intelligence are population-based, stochastic algorithms that simulate groups of natural organisms such as a flock of birds, fish, and whales, a group of bats and butterflies, colonies of ants and bees, etc. These algorithms perform the search process by investigating within the boundaries of previously discovered parts of the search space (exploitation, intensification) and by exploring novel search regions (exploration, diversification)^29^.

Swarm intelligence algorithms have been applied for solving various real-world numerical optimization problems from different domains such as wireless sensor networks (WSNs)^30–33^, cloud and edge computing^34,35^, image thresholding^36^, and many others^37^. The most current and prominent research field is from the domain of hybrid methods between swarm intelligence and machine learning. Researches from this domain have grown rapidly during the past few years and some examples include hyperparameters’ optimization^8,38,39^, feature selection^40^, predicting time series, e.g. the number of COVID-19 cases^41,42^ and ANNs training^43,44^.

## Proposed method

This section first gives details of the SCA metaheuristics. Afterward, observed shortcomings of its basic version are elaborated. Finally, details of the proposed method that overcomes deficiencies of the basic SCA are provided.

### The original SCA method

The inspiration for SCA is taken from the mathematical model of those two important trigonometric functions^12^. Solutions’ positions in the population are updated based on the sine and cosine functions outputs which makes them oscillate around the best solution. These functions return values between − 1 and + 1, which is the mechanism that keeps the solutions fluctuating. An algorithm starts by randomly generating various candidate solutions within the boundaries of the search space in the initialization phase. Exploration and exploitation are controlled differently throughout the execution by random adaptive variables.

Solutions’ position update process is performed in each iteration by using the following equations^12^:

10 $$\begin{aligned} X^{t+1}_i= & {} X^t_i+r_1\cdot sin(r_2)\cdot |r_3 \cdot P^{*t}_i-X^t_i| \end{aligned}$$

11 $$\begin{aligned} X^{t+1}_i= & {} X^t_i+r_1\cdot cos(r_2)\cdot |r_3 \cdot P^{*t}_i-X^t_i| \end{aligned}$$

where $X^t_i$ and $X^{t+1}_i$ denote the current solution’s position in the *i*-th dimension at *t*-th and $i+1$-th iteration, respectively, $r_{1-3}$ are pseudo-randomly generated numbers, the $P^{*}_i$ represents destination point’s position (current best approximation of an optimum) in the *i*-th dimension, while symbol | | denotes the absolute value.

These two equations are used in combination by using control parameter $r_4$:

12 $$\begin{aligned} X^{t+1}_i= {\left\{ \begin{array}{ll} X^{t+1}_i=X^t_i+r_1\cdot sin(r_2)\cdot |r_3 \cdot P^{*t}_i-X^t_i|,\quad r_4<0.5\\ X^{t+1}_i=X^t_i+r_1\cdot cos(r_2)\cdot |r_3 \cdot P^{*t}_i-X^t_i|,\quad r_4\ge 0.5, \\ \end{array}\right. } \end{aligned}$$

where $r_4$ represents a randomly generated number between 0 and 1.

It is noted that for every component of each solution in the population, new values for pseudo-random parameters $r_{1-4}$ are generated.

Four random parameters control the algorithm’s search process and they influence current and the best solution positions. The balance between solutions is required to efficiently converge towards the global optima and it is achieved by changing the range of the based functions in an ad-hoc manner. The sine and cosine functions exhibit cyclic patterns which allow for reposition around the solution. This behavior guarantees exploitation. The algorithm needs to be enabled to search outside of their corresponding destinations which is possible due to the changes in ranges of sine and cosine functions. Furthermore, the solution requires its position not to overlap with the areas of other solutions.

For better quality of randomness, the values for parameter $r_2$ are generated within the range $[0,2\Pi ]$ and that guarantees exploration. The Eq. (13) controls the balance between diversification and exploitation.

13 $$\begin{aligned} r_1=a-t\frac{a}{T}, \end{aligned}$$

where *t* is the current iteration, *T* represents the maximum number of iterations in a run, while *a* is a constant.

### Limitation of basic SCA and proposed hybrid algorithm

The SCA metaheuristics is relatively simple and it does not incorporate many control parameters, yet manages to obtain outstanding results for bound-constrained and constrained benchmarks^12^, as well as for various practical challenges^13^.

Notwithstanding the good exploitation and exploration performance of original SCA, by executing extensive empirical simulations with standard Congress on Evolutionary Computation (CEC) instances, it was observed that in some runs. the algorithm in later iterations converges to the optimal region and does not have enough cycles to perform there a fine-tuned exploitation. This is mainly because the basic search equation (Eq. 12), either by executed with sine, either by cosine operations, is oriented towards the current best approximation of the optimum ($P^{*}_{i}$) for each solutions’ parameter *i*. Moreover, notwithstanding that the basic SCA’s search is very efficient in exploitation, there is still some space for improvements.

Taking into account observed drawbacks of the original SCA, with an expense of increasing algorithms’ complexity, the following changes were incorporated in the basic SCA:

1. the opposition-based learning (OBL) mechanism is applied to current best solution $P^{*}$ and
2. search equation from the well-known FA metaheuristics, that proved excellent exploitation capabilities^45,46^, is used in the basic SCA search process along with sine and cosine search equations.

The OBL mechanism is introduced in^47^ and it was proven that it can substantially enhance the metaheuristics search process. For each *i*-th component of the solution *X*, the opposite individual $X^{o}$ is generated by using Eq. 14.

14 $$\begin{aligned} X^o_i=lb_i+ub_i-x_i \end{aligned}$$

where $lb_i$ and $ub_i$ are lower and upper bounds of component *i*, respectively.

It would be computationally expensive if the OBL is applied to every solution in population in each iteration, so this mechanism is applied only for the current best solution in the following way: in each iteration, the opposite current best solution $P^{*o}$ is created, then if the fitness of $P^{*o}$ is better than the fitness of $P^{*}$, the $P^{*o}$ is designated as the new current best $P^{*}$. In this way, if in earlier iterations the algorithm misses the optimum domain, there are high chances the current’s best opposite would hit the optimum.

On top of the first change, in each iteration the following FA search equation is used with the equal probability along with sine and cosine search mechanisms^48^:

15 $$\begin{aligned} x_{i}^{t+1}=x_{i}^t+\beta _{0} \cdot e^{-\gamma r^{2}_{i,j}}(x_{j}^t-x_{i}^t)+\alpha ^t(\kappa -0.5) \end{aligned}$$

where $x_{i}^{t+1}$ parameter $\beta _{0}$ denotes the attractiveness at a distance $r=0$, $\alpha$ marks a randomization parameter, $\kappa$ represents a random number drawn from either uniform or Gaussian distribution, and $r_{i,j}$ represents the distance between two observed fireflies *i* and *j*. It is noted that contrarily to the original FA, the solution $X_{j}$ is chosen randomly from the population.

The proposed method uses dynamic parameter *alpha* as it was suggested in^48^. In this way, a trade-off between exploitation and exploration is being adjusted in favor of intensification as the iterations progress, as shown in Eq. (16). More details regarding the FA’s control parameters can be captured from^48^.

16 $$\begin{aligned} \alpha ^{(t+1)} = \alpha ^{(t)} \cdot \bigg (1-\dfrac{t}{T}\bigg ) \end{aligned}$$

With the FA’s search equation, the basic SCA equation 12 is updated as follows:

17 $$\begin{aligned} X^{t+1}_i= {\left\{ \begin{array}{ll} \text {apply} \,\, Eq. (10), \quad r_4<0.3\\ \text {apply} \,\, Eq. (11), \quad r_4 \in [0.3,0.6) \\ \text {apply}\,\, Eq. (15), \quad r_4 \ge 0.6 \end{array}\right. } \end{aligned}$$

The proposed method is named opposition best SCA firefly search (OBSCA-FS) and its pseudo-code is given in Algorithm 1.

Some practical limitations of the proposed method include additional control parameters and more function evaluations in each iteration, which is considered in experiments for a fair comparative analysis.

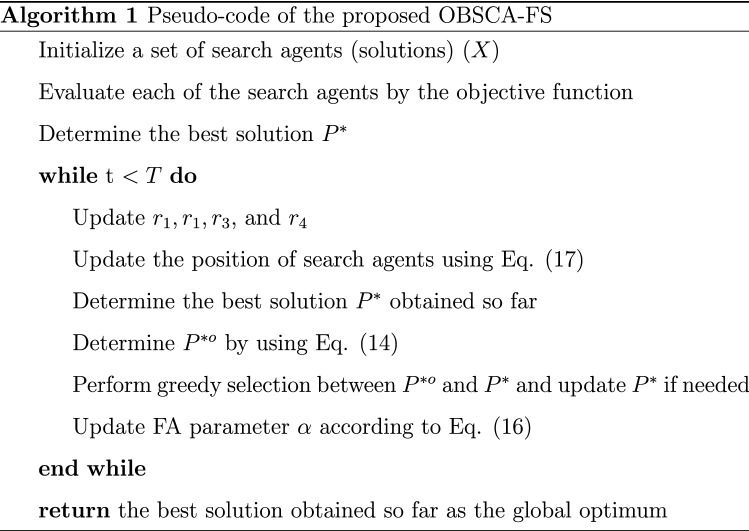



#### The OBSCA-FS complexity

The amount of fitness function evaluations (FFEs) is commonly used as a measure to describe the complexity of the swarm intelligence metaheuristics approaches, due to the fact that the evaluation of the objective function represents the most computationally expensive operation, as discussed by^49^. The complexity is typically expressed in terms of FFE over the number of solutions *N* and number of iterations *T*.

The proposed OBSCA-FS algorithm utilizes just one additional solution evaluation in each iteration during the execution, when the opposite solution of the current best solution is being generated. Consequently, if *N* denotes the number of solutions, and *T* represents the number of iterations, the complexity of the OBSCA-FS can be formulated as $O(N) + O(T\cdot (N+1))$. Thus, the proposed algorithm slightly increases the complexity of the original algorithm.

Moreover, the slight increase in complexity over the basic algorithms is justified because the OBSCA-FS algorithm exhibits significant performance improvements over both basic algorithms, SCA and FA. The enhancements are obvious both for benchmark function set and for the dropout regularization machine learning task, as described in “CEC2019 benchmark simulations” and “CNN dropout regularization simulations” Sections.

### Ethics approval

The authors declare that they their work is compliant with ethical standards.

### Consent to participate

All authors have given their consent for this research.

### Consent for publication

All authors have given their consent for publication of this work.

## CEC2019 benchmark simulations

Following proper experimental design, the proposed method was first validated on standard unconstrained benchmarks before it is applied for a real-world problem. In the case of devised OBSCA-FS, a group of 10 relatively novel Congress on Evolutionary Computation 2019 (CEC2019)^50^ benchmarks was utilized and simulation results along with comparative analysis and statistical tests are presented in this section.

The results of the proposed OBSCA-FS are compared to the original SCA and FA, and other eight state-of-the-art metaheuristics (EHOI, EHO, SSA, GOA, WOA, BBO, MFO, PSO). For the purpose of this research, all above mentioned approaches were implemented and tested. Results of all opponents, including the original SCA, were obtained through extensive simulations. In an effort for this research, original FA was also implemented and tested.

Experimental results for the same set of benchmark functions were previously reported in^51^. Nevertheless, simulations presented in this research have been recreated to validate results from^51^ and to provide firm grounds for a more objective comparative analysis. Algorithms in^51^ were executed by using *N*=50 and *T*=500, and this experimental setup could result in biased comparative analysis due to the fact that not all methods use the same amount of *FFEs* in one iteration. To solve this potential problem, this research utilizes the termination condition that was set according to the total used *FFEs* for all observed methods. To establish similar experimental preconditions as in^51^, the *maxFFEs* number was set to 25.000 ($50\cdot 500$).

The summary of OBSCA-FS control parameters’ adjustments used in experiments is shown in Table 1. The same control parameters were used also in FA’s evaluations.

**Table 1:** Control parameter summary.

**Parameter description**	**Value**
Population size *N*	49
Maximum iteration number *T*	500
Parameter $r_1$	Changes according to Eq. (13)
Parameter $r_2$	$\in [0,2\pi$]
Parameter $r_3$	$\in [0,1]$
Parameter $r_4$	$\in [0,1]$
FA’s Absorption coefficient $\gamma$	1
FA’s attractiveness parameter at $r=0$ $\beta _{0}$	1
FA’s randomization parameter $\alpha$	Changes according to Eq. (16)
FA’s Initial $\alpha$ value $\alpha _{0}$	0.5

Obtained experimental results and comparison for mean and standard deviation values for 10 CEC2019 functions are provided in Table 2, where for better readability best results for the mean indicator are marked bold.

**Table 2:** Result comparison of different well-known metaheuristics on CEC2019 benchmark functions.

Function	Stats	EHOI	EHO	SCA	SSA	GOA	WOA	BBO	MFO	PSO	FA	OBSCA-FS
CEC01	Mean	4.76E+04	1.35E+07	9.83E+09	3.21E+09	1.61E+10	1.03E+10	3.52E+10	7.17E+09	6.75E+11	7.43E+04	**4.83E+03**
	Std	2.14E+03	7.91E+06	6.95E+09	1.42E+09	8.99E+9	9.14E+09	2.32E+10	8.69E+09	2.34E+11	4.49E+03	4.21E+03
CEC02	Mean	1.70E+01	1.72E+01	1.75E+01	1.73E+01	1.74E+01	1.73E+01	8.87E+01	1.74E+01	8.56E+02	2.85E+01	**2.41E+00**
	Std	3.66E−16	7.29E−15	5.19E−03	6.55E−05	3.23E−02	1.95E−03	2.45E+01	4.17E−15	3.87E+02	3.21E+02	5.32E+01
CEC03	Mean	1.27E+01	1.27E+01	1.27E+01	1.27E+01	1.27E+01	1.27E+01	1.27E+01	1.27E+01	1.27E+01	1.27E+01	1.27E+01
	Std	3.95E−16	7.44E−16	3.25E−04	3.11E−15	6.47E−04	7.94E−06	5.25E−07	4.38E−05	4.12E−04	5.22E−01	4.03E−01
CEC04	Mean	1.28E+01	1.55E+01	8.32E+02	3.25E+01	1.51E+02	2.65E+02	6.95E+01	1.38E+02	6.92E+01	3.89E+01	**1.00E+01**
	Std	4.26E+00	8.52E+00	3.85E+02	1.09E+01	1.13E+02	1.39E+02	2.99E+01	1.15E+02	5.43E+01	2.32E−01	1.19E+00
CEC05	Mean	1.05E+00	1.07E+00	2.23E+00	1.35E+00	1.33E+00	1.67E+00	1.31E+00	1.13E+00	1.55E+00	1.13E+00	**1.01E+00**
	Std	3.25E−03	2.41E−02	7.81E−02	2.33E−01	1.21E−01	3.86E−02	9.63E−02	6.56E−02	1.18E−01	4.26E−02	2.17E−02
CEC06	Mean	8.33E+00	9.45E+00	1.04E+01	3.79E+00	6.19E+00	9.14E+00	5.78E+00	4.92E+00	1.03E+01	1.05E+01	**1.86E+00**
	Std	6.23E−01	1.31E+00	8.15E+00	1.23E+00	1.33E+00	1.05E+00	2.99E−01	2.13E+00	3.35E+00	6.20E−01	4.46E−02
CEC07	Mean	1.42E+02	1.81E+02	6.38E+02	2.89E+02	2.87E+02	4.53E+02	4.92E+00	3.19E+02	5.97E+02	4.91E+02	**3.85E+00**
	Std	1.13E+02	1.51E+02	2.78E+02	2.25E+02	1.75E+02	2.25E+02	1.21E+00	2.15E+02	1.89E+02	1.23E+02	8.36E+01
CEC08	Mean	**2.69E+00**	3.15E+00	5.77E+00	5.08E+00	5.49E+00	5.75E+00	4.81E+00	5.45E+00	5.10E+00	2.78E+00	2.83E+00
	Std	9.15E−02	1.44E+00	7.29E−01	7.83E−01	5.14E−01	7.29E−01	1.03E+00	5.62E−01	7.33E−01	8.99E−01	9.13E−01
CEC09	Mean	2.29E+00	2.41E+00	8.75E+01	2.38E+00	2.45E+00	5.16E+00	3.75E+00	2.46E+00	2.65E+00	4.95E+00	**1.73E+00**
	Std	5.55E−03	2.18E−02	5.63E+01	5.33E−02	6.41E−02	5.29E−01	3.14E−01	6.76E−02	8.45E−02	2.83E−01	1.54E−02
CEC10	Mean	1.92E+01	2.11E+01	2.08E+01	2.03E+01	2.00E+01	2.05E+01	2.07E+01	2.02E+01	2.06E+01	2.02E+01	**1.32E+01**
	Std	3.49E+00	7.29E+00	6.45E+00	8.19E+00	6.67E+00	3.52E−01	7.13E−00	6.66E−01	9.81E+02	9.13E−02	1.56E−02

Reported results in Table 2 communicate the superior performance of proposed OBSCA-FS when compared to other state-of-the-art approaches. For almost all instances, the OBSCA-FS managed to establish the best mean value. The only exceptions are CEC03 benchmark, where all approaches obtained the same mean indicator value and CEC08 in which case the results reported for EHOI are the best, while original FA performed slightly better than OBSCA-FS. Furthermore, based on experimental data it can be concluded that the OBSCA-FS hybrid method substantially improves the performance of both algorithms, SCA and FA, which is at the same time the basic goal of devising hybrid methods.

However, when comparing different methods it is not enough just to state that one method is better than the other in terms of results, it also should be determined whether the enhancements are statistically significant. Following this assumption, a Friedman test^52,53^ and two-way analysis of variance by ranks is performed to validate the significant difference between proposed OBSCA-FS and other adversary metaheuristics. The Friedman test results for eleven approaches applied to 10 functions are provided in Table 3.

**Table 3:** Friedman ranks for the comparable method over 10 CEC2019 instances.

Function	EHOI	EHO	SCA	SSA	GOA	WOA	BBO	MFO	PSO	FA	OBSCA-FS
CEC01	2	4	7	5	9	8	10	6	11	3	1
CEC02	4	4	8	4	7	4	10	4	11	9	1
CEC03	6	6	6	6	6	6	6	6	6	6	6
CEC04	2	3	11	4	9	10	7	8	6	5	1
CEC05	2.5	2.5	11	6	8	10	7	5	9	4	1
CEC06	6	8	11	2	5	7	4	3	10	9	1
CEC07	3	4	11	5	6	8	2	7	10	9	1
CEC08	1	4	11	7	8	10	5	9	6	2	3
CEC09	2	3	11	4	5	9	8	6	7	10	1
CEC10	2	9	11	3	4.5	7	4.5	7	10	7	1
Average	3.05	4.75	9.8	4.6	6.75	7.9	6.35	6.1	8.6	6.4	1.7
Rank	2	4	11	3	8	9	6	5	10	7	1

From Table 3 can be noticed that the proposed OBSCA-FS is advanced in terms of performance than the other 10 algorithms with an average rank of 1.7. The basic FA and SCA have average rankings of 6.4 and 9.8, respectively. Also, the Friedman statistics ($\chi ^2_r = 51.2$) is greater than the $\chi ^2$ critical value with 10 degrees of freedom (18.3), at significance level $\alpha = 0.05$, therefore the null hypothesis ($H_0$) is rejected and it can be concluded that the proposed OBSCA-FS obtains results which are significantly different than other 10 methods.

However, in^54^ is reported that the Iman and Davenport’s test^55^ may be more precise than the $\chi ^2$ having this in mind, Iman and Davenport’s test was also executed. Calculated statistic of $9.46E+00$, which is greater than the *F*-distribution critical value ($F(9,9\times 10) = 1.93E+00$). Therefore, Iman and Davenport’s test also rejects $H_0$. In the case of both tests, the $p-value$ is less than the significance level. Summary of results for both statistical tests is given in Table 4.

**Table 4:** Friedman and Iman-Davenport test results ($\alpha =0.05$).

Friedman value	$\chi ^2$ critical value	*p*-value	Iman-Davenport value	*F* critical value	*p*-value
5.12E+01	1.83E+01	1.11E−16	9.46E+00	1.93E+00	1.11E−13

As the null hypothesis is rejected by Friedman and Iman and Davenport tests, we proceeded with the non-parametric post-hoc procedure, with Holm’s step-down procedure, and the obtained results are reported in Table 5.

**Table 5:** Holm’s step-down procedure result.

Comparison	*p*-value	Rank	0.05/(k-i)	0.1/(k-i)
OBSCA-FS versus SCA	2.37E−08	0	0.005000	0.01000
OBSCA-FS versus PSO	1.46E−06	1	0.005556	0.01111
OBSCA-FS versus WOA	1.46E−05	2	0.006250	0.01250
OBSCA-FS versus GOA	3.31E−04	3	0.007143	0.01429
OBSCA-FS versus FA	7.66E−04	4	0.008333	0.01667
OBSCA-FS versus BBO	8.59E−04	5	0.010000	0.02000
OBSCA-FS versus MFO	1.50E−03	6	0.012500	0.02500
OBSCA-FS versus EHO	1.98E−02	7	0.016667	0.03333
OBSCA-FS versus SSA	2.52E−02	8	0.025000	0.05000
OBSCA-FS versus EHOI	1.81E−01	9	0.050000	0.10000

Table 5 shows that the proposed method significantly outperformed all compared methods at significance level $\alpha =0.1$, as well as all algorithms except EHOI at significance level $\alpha =0.05$.

**Figure 1:**
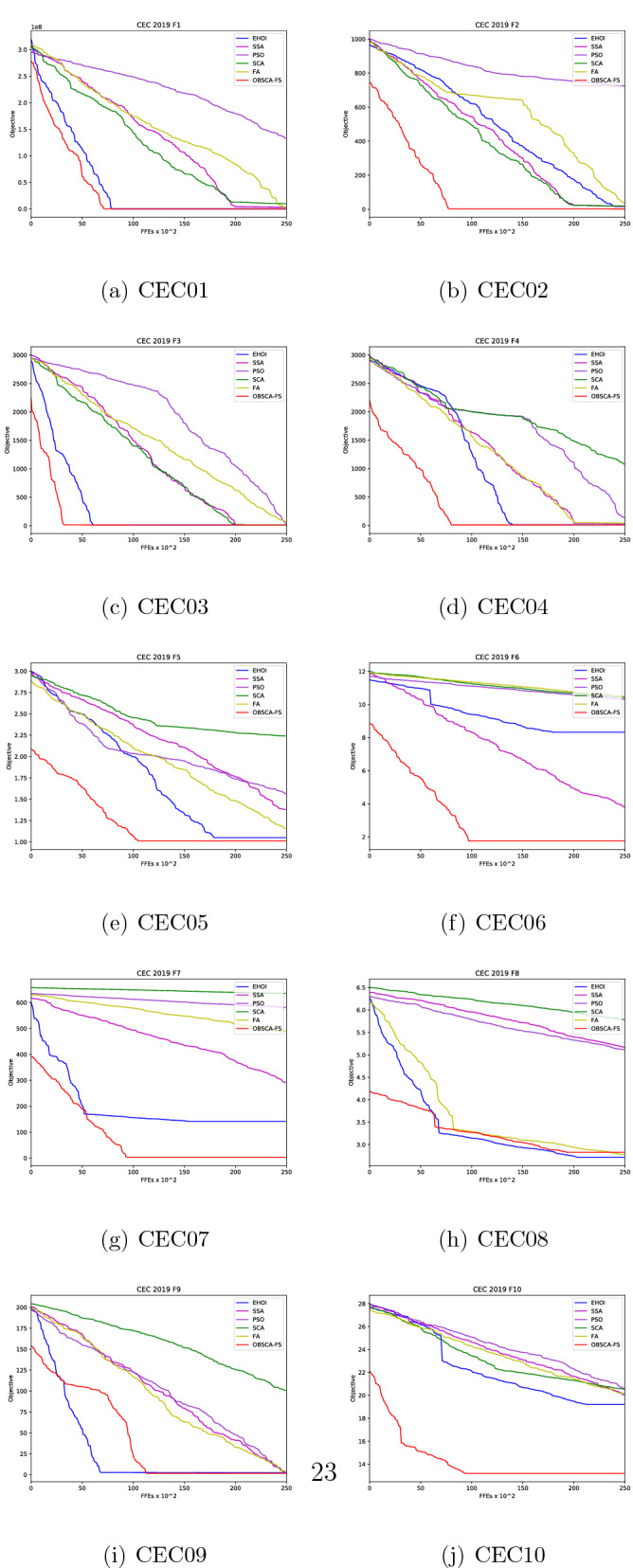
Convergence speed graphs of the 10 CEC 2019 benchmark functions as direct comparison between proposed OBSCA-FS and relevant metaheuristics.

Figure 1 illustrates a head-to-head convergence comparison between the proposed OBSCA-FS, second best approach (EHOI) and relevant basic metaheuristics, with respect to the results on the 10 benchmark functions. Since OBSCA-FS is a hybrid of SCA and FA, convergence graphs for those two methods were included as well. The plots illustrate how the fitness evaluation decreases over the course of iterations for each test function in turn.

It is important to notice from the convergence graphs that the proposed algorithm combines the best elements of the FA and SCA metaheuristics. It is obvious from the Fig. 1 that OBSCA-FS converges faster from both FA and SCA, therefore establishing itself as more efficient metaheuristics. However, according to the no free lunch theorem, there is no general metaheuristics that will be a perfect solution for all problems. This can be seen on convergence graph for the benchmark F8, where basic FA obtained better results than the proposed OBSCA-FS. Nevertheless, this behavior is normal and expected, as there is always a trade-off.

## CNN dropout regularization simulations

This section is divided into two parts. In the first part, research findings and comparative analysis from experiments with four standard benchmark datasets are shown, while the second part provides details for dropout regularization experiments with specific MRI dataset.

### Dropout simulations for benchmark datasets

The model used in conducted experiments is the same as the one proposed in the referenced paper^7^, where the performance of distinguished bat algorithm (BA), cuckoo search (CS), FA, and particle swarm optimization (PSO) swarm intelligence metaheuristics for dropout regularization challenge was reported. In this manuscript, the same experimental environment as in^7^ was established due to the fair comparative analysis. As acclaimed in “Introduction” Section, the potential of swarm intelligence approaches for this problem was not fully investigated and the aim of this experiment is to further examine its potential in this domain, as well as to validate the proposed state-of-the-art hybrid OBSCA-FS method against this practical challenge.

The testing framework was developed in Python with its core and data science libraries and API’s keras, scikitlearn, numpy, scipy along with pandas and matplotlib for visualization. The machine that was used for testing has a 6 $\times$ NVIDIA GTX 1080 GPU with Intel$\textregistered$ CoreTM i7-8700K CPU, 64GB RAM, and Windows 10 OS.

For validation, 4 standard datasets were used: MNIST (http://yann.lecun.com/exdb/mnist/), Semeion (https://archive.ics.uci.edu/ml/datasets/Semeion+Handwritten+Digit), USPS (http://statweb.stanford.edu/tibs/ElemStatLearn/datasets/zip.info.txt) and CIFAR10 (http://www.cs.toronto.edu/kriz/cifar.html). For more details regarding characteristics and the number of available observations in each dataset, please refer to the provided links.

**Figure 2:**
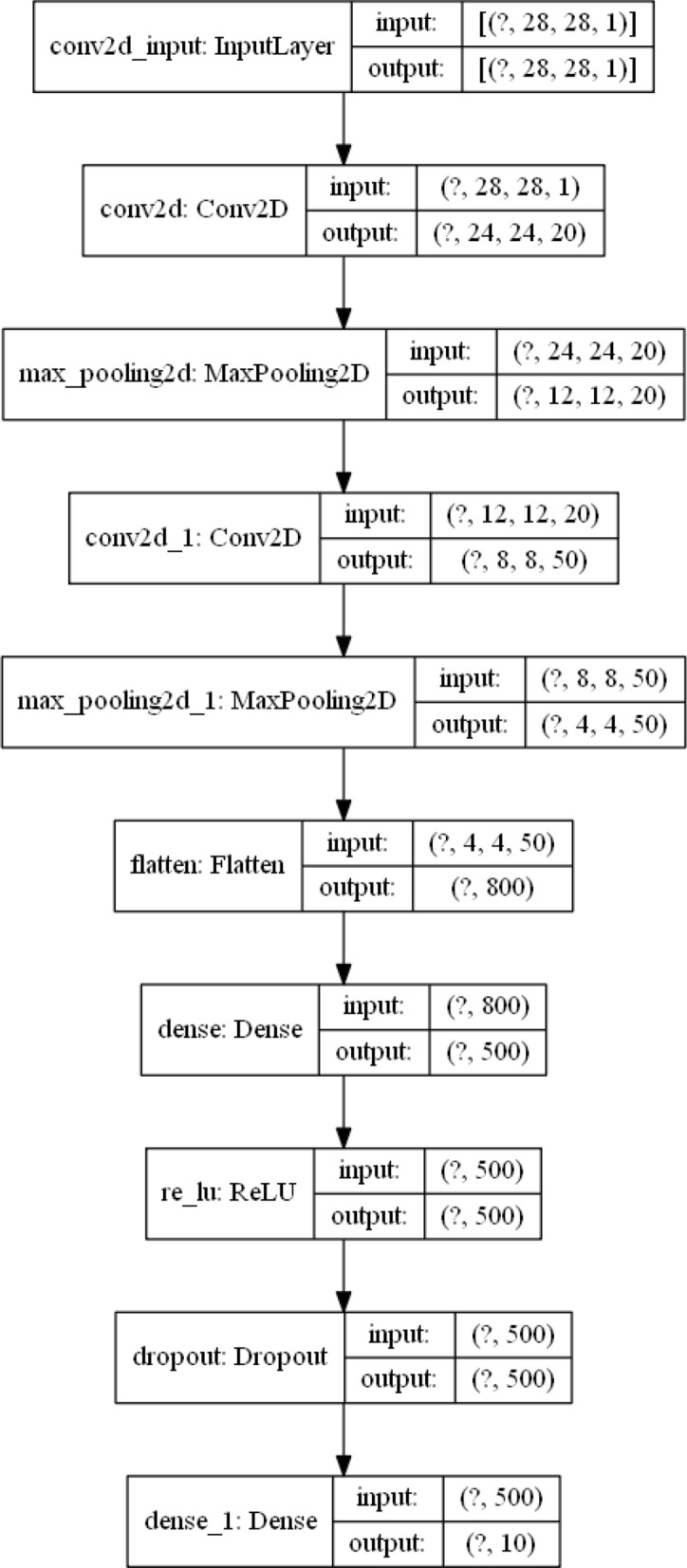
Example instance of MNIST, Semeion and USPS model.

**Figure 3:**
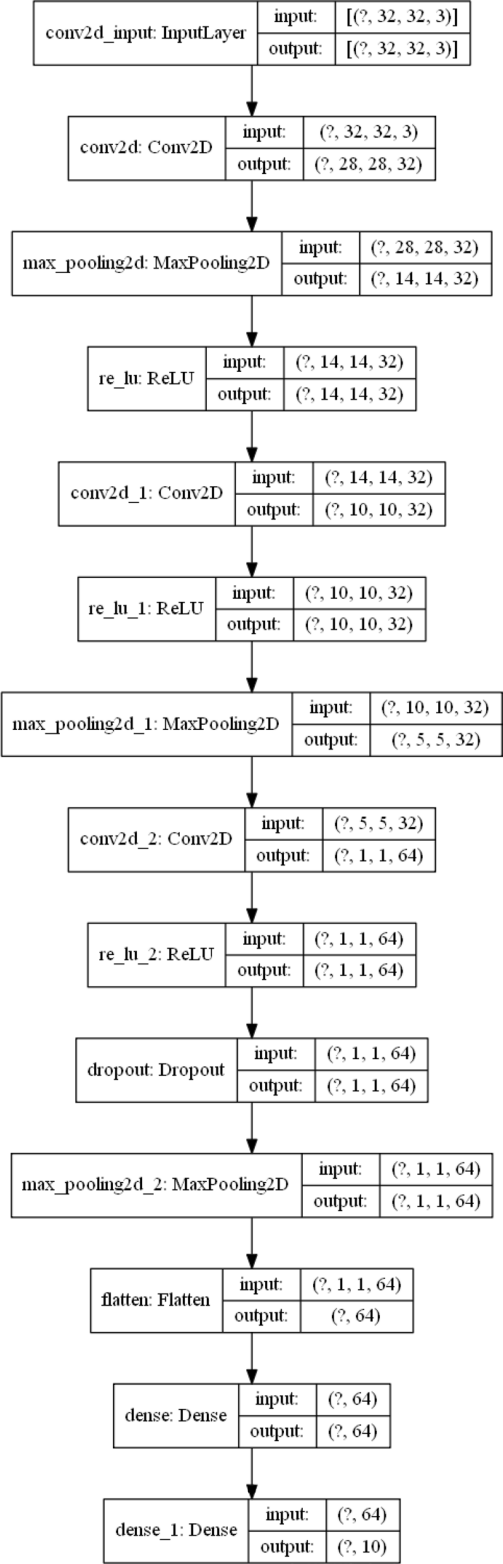
Example instance of CIFAR10 model.

Two different CNN architectures as in^7^, as it is provided in the default Caffe examples, are utilized: one for MNIST, Semeion, and USPS and one for the CIFAR-10 dataset. The only difference is that in both models additional dropout layer is added and in the case of USPS and Semeion datasets, the kernel size of $3\times 3$ instead of $5\times 5$ is used for convolution layers because these two datasets have lower resolution. Example instances of two models are given in Figs. 2 and 3.

In all simulations besides the dropout probability (*dp*) for the dropout layer, which is subject to optimization, L1 regularization (penalty) $\alpha$ and L2 regularization (weight decay) $\lambda$ were also employed. For training the models, RMSProp optimizer was executed with a learning rate $\eta$. The parameters’ tuple ($\eta$, $\alpha$, $\lambda$) was fixed and only *dp* was optimized. Therefore, solutions’ encoding is very straightforward and every solution consists of only one parameter with values $\in [0,1]$.

**Figure 4:**
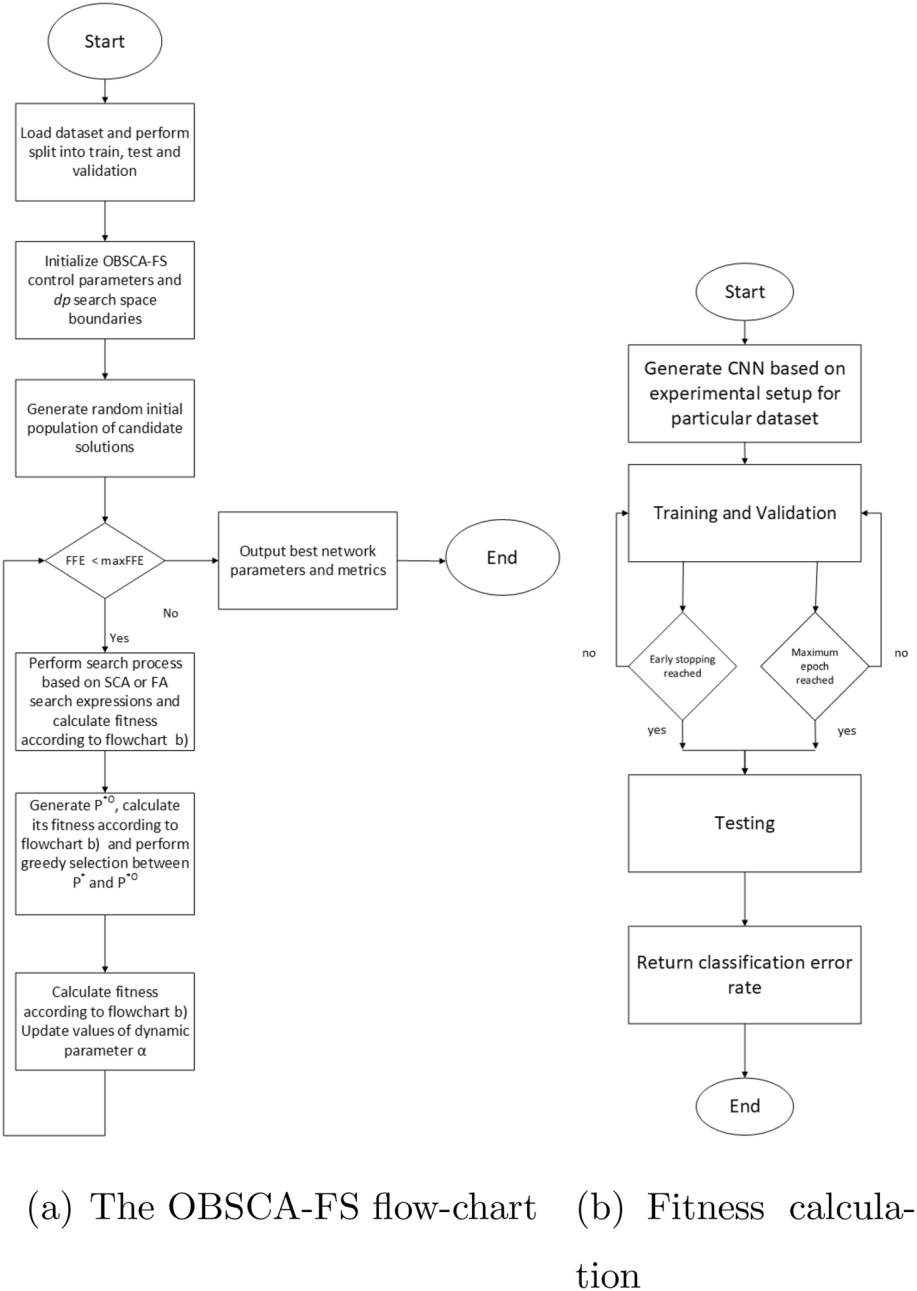
Proposed methodology for *dp* regularization.

The classification error rate is used as the fitness function, therefore a problem is formulated as a minimization challenge. With the goal of visualizing proposed methodology, general OBSCA-FS and fitness calculation flow-charts are shown in Fig. 4.

Additionally, with the goals of straightforward comparative analysis, in the context that all metaheuristics are tested under the same conditions, and of establishing referenced (baseline) models, standard Caffe architecture with and without dropout is also included in comparison and for other metaheuristics, standard default Caffe parameters for $\eta$, $\alpha$, and $\lambda$ were used. These parameters over utilized datasets are summarized in Table 6.

**Table 6:** The CNN $\eta$, $\alpha$ and $\lambda$ adjustments for simulations.

Dataset	$\eta$	$\alpha$	$\lambda$	*dp*
CIFAR-10	0.001	0.9	0.004	[0, 1]
MNIST	0.01	0.9	0.00005	[0, 1]
Semeion	0.001	0.9	0.00005	[0, 1]
USPS	0.01	0.9	0.00005	[0, 1]

All datasets were split into the training, validation, and testing sets. For determining the fitness of each individual, the classification accuracy of the test set is used. In all experiments, the $categorical_cross_entropy$ loss function is employed and for the CIFAR-10 dataset model is trained in 4,000, while for the remaining 3 datasets, 10,000 epochs are used for training. Model details and the number of samples along with the batch size are summarized in Table 7.

**Table 7:** Configuration of the datasets used in experiments.

Dataset	Training set samples (batch size)	Validation set samples (batch size)	Testing set (batch size)	Epochs
CIFAR-10	20,000 (100)	30,000 (100)	10,000 (100)	4000
MNIST	20,000 (64)	40,000 (100)	10,000 (100)	10,000
Semeion	200 (2)	400 (400)	993 (993)	10,000
USPS	2406 (32)	4885 (977)	2007 (2007)	10,000

For the purpose of this experiment, all approaches that were included in the comparative analysis were implemented and tested. Control parameters for BA, FA, CS, and PSO that were used in the comparative analysis can be retrieved from^7^. Proposed OBSCA-FS was tested with the same parameters as presented in Table 1. Moreover, as already stated, to further investigate swarm algorithms’ performance for tackling dropout regularization issue, the EHO, WOA, SSA, GOA, BBO and SCA metaheuristics were also implemented and tested with the suggested parameters for bound-constrained optimization in relevant publications. The control parameters values are summarized in Table 8. All metaheuristics approaches were executed with a total number of 77 *FFEs*. The study proposed in^7^ evaluated methods with $N=7$ and $T=10$, which also yielded a total of 77 *FFEs* (7 + 7 $\times$ 10). Cross-validation with 20 runs was carried out for the purpose of providing statistical Friedman’s non-parametric test and average results over 20 independent runs are reported.

**Table 8:** Configuration of control parameters of the metaheuristics that were implemented and included in the comparative analysis.

Algorithm	Control parameters and their values
BA^56^	$f_{min} = 0$, $f_{max} = 2$, $A = 0.5$, $rand = 0.5$
CS^57^	$\beta = 1.5$, $p = 0.25$, $\alpha = 0.8$
PSO^58^	$c_1 = 1.7$, $c_2 = 1.7$, $w = 0.7$
EHO^59^	$no_clan = 5$, $\alpha = 0.5$, $\beta =0.1$, $no_elite = 2$
WOA^60^	Initial value of $a = 2.0$, linearly decreasing to 0
SSA^61^	$c_{1}$ non-linearly decreasing from 2 to 0, *c*2 and *c*3 rand from [0,1]
GOA^62^	*c* linearly decreasing from 1 to 0
BBO^63^	$hmp= 1$, $imp= 0.1$, $nbhk = 2$
FA^48^	$\gamma = 1.0$, $\beta _0 = 1.0$, $\alpha = 0.2$
SCA^12^	$a=2$, $r_{1}$ linearly decreasing from 2 to 0

The average value of accuracy along with the mean value of *dp* obtained in the MNIST, Semeion, USPS and CIFAR-10 datasets simulations are reported in Table 9. Best obtained accuracy among metaheuristics-based approaches is marked bold. From the presented table, it can be seen that the average accuracy is not consistent over the observed datasets. This is mostly because of the different and distinctive nature of each dataset included in the experiments, in terms of the overall number of images in the dataset, the amount of features, and the contents of images.

**Table 9:** Comparative results of the suggested OBSCA-FS method and other metaheuristics approaches in terms of mean classification accuracy.

Method	MNIST		Semeion		USPS		CIFAR-10	
	acc.	*dp*	acc.	*dp*	acc.	*dp*	acc.	*dp*
Caffe	99.07	0	97.62	0	95.80	0	71.47	0
Dropout Caffe	99.18	0.5	98.14	0.5	96.21	0.5	72.08	0.5
BA	99.14	0.491	98.35	0.692	96.45	0.762	71.49	0.633
CS	99.14	0.489	98.21	0.544	96.31	0.715	71.21	0.669
PSO	99.16	0.493	97.79	0.371	96.33	0.725	71.51	0.621
EHO	99.13	0.475	98.11	0.481	96.24	0.682	71.15	0.705
WOA	99.15	0.489	98.23	0.561	96.32	0.722	71.23	0.685
SSA	99.19	0.499	98.31	0.642	96.41	0.753	71.58	0.529
GOA	99.16	0.492	98.15	0.513	96.15	0.481	70.95	0.849
BBO	99.13	0.474	98.16	0.515	96.17	0.483	71.08	0.768
FA	99.18	0.495	98.29	0.619	96.42	0.758	71.55	0.583
SCA	99.17	0.496	98.25	0.580	96.29	0.705	71.54	0.597
OBSCA-FS	**99**.**28**	0.524	**98**.**48**	0.722	**96**.**93**	0.838	**72**.**54**	0.394

The findings presented in Table 9 point out the superior performance of the suggested OBSCA-FS approach in regard to the *dp* value that has been subjected to the optimization. On the MNIST dataset, the suggested OBSCA-FS approach achieved the superior accuracy of 99.28% with obtained *dp* value of 0.524. On the same dataset, other metaheuristics algorithms determined the *dp* value below the value of the standard Dropout Caffe $dp=0.5$. In this scenario, the findings indicate that the *dp* value should be just over 0.5 to obtain better accuracy values, and the suggested OBSCA-FS approach was the only algorithm that has achieved it.

On the Semeion dataset, the suggested OBSCA-FS algorithm achieved the best accuracy of 98.48%, by obtaining the *dp* value of 0.722. In this case, it can be concluded that the accuracy increases with values of *dp*, that are over the standard Dropout Caffe value 0.5. The runner-up approach was BA, that was able to achieve the accuracy of 98.35% by $dp=0.692$. The basic Caffe method that does not utilize the dropout ($dp=0$) was able to achieve 97.62% accuracy, while the Dropout Caffe ($dp=0.5$) obtained the accuracy of 98.14%.

Similar results are observed on the USPS dataset as well. The suggested OBSCA-FA method again obtained the best accuracy of 96.93% with determined *dp* value of 0.838. Similarly to the first two observed datasets, the accuracy increases with the increase of *dp* value. The second best result has been obtained by BA, that achieved 96.45 % by $dp=0.762$. The basic Caffe and Dropout Caffe were significantly behind the proposed OBSCA-FA, with the accuracy lesser by approx. 1.1% and 0.7%, respectively.

Lastly, in the case of CIFAR-10 dataset, a different pattern can be observed. The findings indicate that, if the *dp* is greater than the Dropout Caffe ($dp=0.5$), the performance starts to decrease, while the accuracy drops down. In other words, the network drops out neurons, and it starts to lose the ability to generalize well. On the other side, if the *dp* value is too small, again, the accuracy will decrease (similar to the basic Caffe with $dp=0$). The best performances for the CIFAR-10 dataset are achieved for the *dp* values slightly lesser than 0.5. The proposed OBSCA-FA approach scored the best accuracy of 72.54% with the $dp=0.394$. Moreover, it was the only approach that was able to find the *dp* value below 0.5 boundary, while other metaheuristics approaches got stuck with the *dp* values in range [0.5, 1].

Finally, it is worth noticing that the OBSCA-FS approach, as a hybrid between SCA and FA, significantly outperformed both basic metaheuristics versions in all performed tests. This way, the enhancements of the OBSCA-FS over the original implementations of SCA and FA, observed on the unconstrained benchmark functions tests, were also confirmed on the practical task of optimizing the dropout regularization.

**Figure 5:**
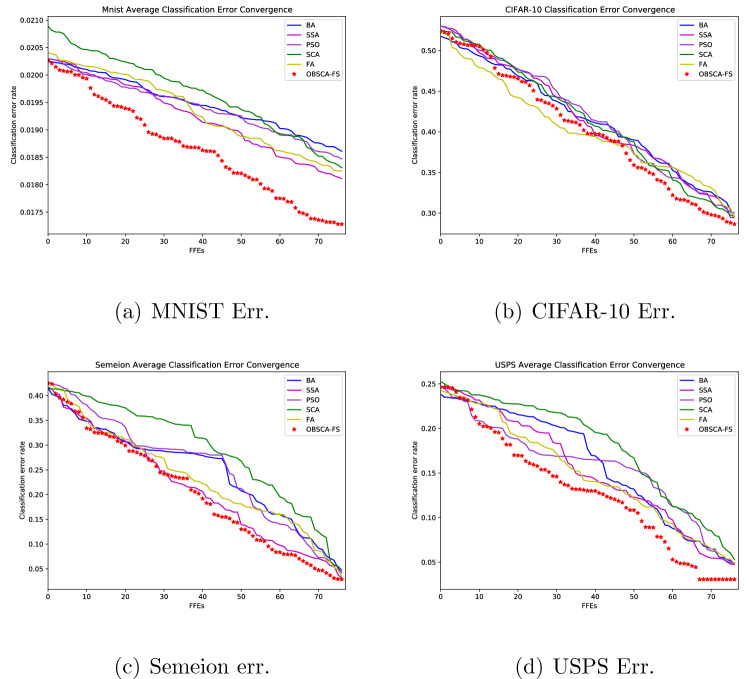
Convergence graph of MNIST, CIFAR-10, Semeion and USPS datasets for average classification error for OBSCA-FS, SCA and WOA.

Average convergence speed graphs for implemented OBSCA-FS, SCA, FA and three other representative metaheuristics (PSO, BA and SSA) for mean classification error for MNIST, CIFAR-10, Semeion, and USPS datasets, generated over 20 independent runs with 77 FFEs, are provided in Fig. 5.

Similarly, as in unconstrained experiments, a Friedman test and two-way analysis of variance by ranks are performed. Results are shown in Table 10. From presented statistical tests can be observed that the proposed OBSCA-FS established with the rank 1, the SSA proved as the second-best metaheuristics, while the rank of 3 obtained the original FA metaheuristics.

After completing the necessary calculations, the Iman and Davenport test score was 36.95, and it was compared to the *F*-distribution critical value ($F(9,9\times 10) = 1,820$). As the test returns considerably larger value, this test rejects $H_0$. Additionally, the Friedman statistics ($\chi ^2_r = 181,50$) is greater than the $\chi ^2$ critical value with ten degrees of freedom (1, 82), with the significance level $\alpha = 0.05$.

Finally, the null hypothesis ($H_0$) can be rejected. This indicates that OBSCA-FS performances were significantly superior over the rest of the metaheuristics included in the experiments.

**Table 10:** Friedman ranks for the comparable method over 4 CNN classification instances.

Function	Caffe	Dropout Caffe	BA	CS	PSO	EHO	WOA	SSA	GOA	BBO	FA	SCA	OBSCA-FS
MNIST	13	3.5	9.5	9.5	6.5	11.5	8	2	6.5	11.5	3.5	5	1
Semeion	13	10	2	7	12	11	6	3	9	8	4	5	1
USPS	13	10	2	7	5	9	6	4	12	11	3	8	1
CIFAR-10	8	2	7	10	6	11	9	3	13	12	4	5	1
Average	11.75	6.375	5.125	8.375	7.375	10.625	7.25	3	10.125	10.625	3.625	5.75	1
Rank	13	6	4	9	8	11	7	2	10	12	3	5	1

Lastly, although not being the main topic of this research described in this paper, another mini experiment has been conducted, to further evaluate the performances of the proposed OBSCA-FS algorithm. In this additional experiment, all four parameters from the Table 6, namely *dp* (dropout probability), $\eta$ (learning rate), $\alpha$ (L1 regularization) and $\lambda$ (L2 regularization - weight decay), have been subjected to the optimization process, without taking the standard default Caffe parameter values. The parameter ranges that were used in the experiments were $dp = [0,1]$, $\eta = [0,1]$, $\alpha = [0,1]$ and $\lambda = [0,1]$.

The experiment utilized the same setup, the same number of runs and the same amount of training as the main benchmark experiments described above. The accuracy results obtained by the basic SCA were as follows: 99.19% on the MNIST dataset, 98.28% on the Semeion dataset, 96.33% on the USPS dataset, and 71.56% on the CIFAR-10 dataset. On the other hand, the accuracy values achieved by the proposed OBSCA-FS method were as follows: 99.32% in the case of MNIST dataset, 98.55% on the Semeion dataset, 97.03% on the USPS dataset, and finally, 72.69% on the CIFAR-19 dataset. The improvement of the accuracy results for both the basic SCA and the proposed OBSCA-FS methods are minor, leading to the conclusion that the default values for $\eta$, $\alpha$ and $\lambda$ used by the Caffe are well adjusted. However, in case of all four parameters being subjected to the optimization process, every metaheuristics solution is being encoded with four continuous parameters. This significantly increases the complexity of the method, while the minor improvements of the accuracy are not justifying it.

### Dropout simulations for MRI benchmark dataset

In a similar way as presented in^64^, MRI dataset was used to further validate the proposed approach. The benchmark dataset (https://figshare.com/articles/dataset/brain_tumor_dataset/1512427) is comprised of 3064 T1-weighted MRIs with Glioma, Meningioma, and Pituitary brain tumor classes, obtained from 233 subjects by Cheng et. al^65^. This research employed the same image processing as in^64^: pixel values for all images were normalized to scale [0, 1], followed by the data augmentation approach to increase the volume of the training set. The dimensions of images were set to $128\times 128$ pixels. Data augmentation step included the generation of fake data by randomly modifying the original images, and the addition of this generated data to the initial dataset. In this research, random modifications included rotating the original image by 10, 20, or 30 degrees in a random direction, translating the image by 15 pixels, resizing the original image, mirroring, and finally, using combinations of modifications at once.

The dataset was originally comprised of 3064 axial images divided into three classes: 708 meningioma, 1426 glioma, and 930 pituitary tumor images. Upon completion of the data augmentation process, every category consisted of 1521 images that were utilized for training phase, and 115 that were used for the testing phase, with the total amount of images being 4908. Additional information about the image pre-processing and dataset splitting can be obtained from^64^.

The same fitness function formulation as in simulations for standard datasets is used and for more details refer to “Dropout simulations for benchmark datasets” Section.

**Figure 6:**
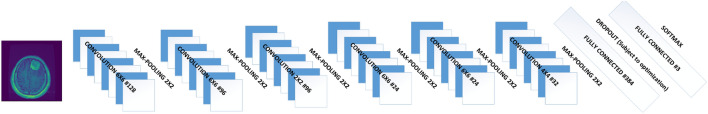
The CNN structure utilized for MRI dataset.

The proposed OBSCA-FS algorithm was tested for the dropout rate and validated against several cutting-edge metaheuristics approaches. The utilized CNN topology was derived from previous research published in^66^, that was dealing with the hyperparameters’ optimization for this particular dataset. The CNN structure shown in Fig. 6 obtained the best results in^66^, and it was consequently utilized in this research for testing and optimizing the dropout probability parameter. Finally, the CNN uses the Adam optimizer and the learning rate $lr=0.0005$, again as the product of research^66^.

Research published in^64^ is evolving 50 ($N=50$) candidate CNN structures in 15 rounds $T=15$, that for most metaheuristics algorithms corresponds to a total number of 800 *FFEs* ($N + N\cdot T$). However, due to the fact that not all metaheuristics algorithms utilize the same amount of *FFEs* in every round of execution, in the proposed research $maxFFEs=800$ was used as the terminating condition. The control parameters for opposing metaheuristics approaches were obtained from the original publications and are shown in Table 11.

The proposed OBSCA-FS driven CNN was also compared to other cutting-edge, non-metaheuristics approaches, including SVM + RFE, Vanilla preprocessing + shallow CNN, LeNet-5, VGG19 and DenseNet. Since those models are considered to be state of the art, they were tested with default parameters.

**Table 11:** Control parameters’ values for metaheuristics methods included in the experiments.

Metaheuristics	Parameters’ values
GA^64^	$p_{c} = 0.5$, $p_{m} = 0.2$
FA^48^	$\alpha = 0.5$, $\beta = 0.2$, $\gamma = 1.0$
mFA^39^	$\alpha = 0.5$, $\beta = 0.2$, $\gamma = 1.0$, $TL = 20 \, FFEs$
BA^56^	$Q_{min} = 0$, $Q_{max} = 2$, $A=0.5$, $r=0.5$
EHO^59^	$no_clan = 5$, $\alpha = 0.5$, $\beta =0.1$, $no_elite = 2$
WOA^60^	$a_{1}$ linearly decreasing from 2 to 0, $a_{2}$ linearly decreasing from -1 to -2, *b*=1
SCA^12^	$a=2$, $r_{1}$ linearly decreasing from 2 to 0

**Table 12:** MRI tumor grades classification comparative analysis.

Approach	Accuracy (%)	Dropout
SVM + RFE^67^	71.2	
Vanilla preprocessing + shallow CNN^68^	91.4	
CNN LeNet-5^69^	74.9	
VGG19^70^	92.6	
DenseNet^71^	92.7	
CNN + GA^64^	94.9	0.33
CNN + mFA^39^	96.9	0.39
CNN + BA^56^	95.6	0.37
CNN + EHO^59^	94.8	0.31
CNN + WOA^60^	95.5	0.36
CNN + HHO^66^	96.5	0.38
CNN + eHHO^66^	98.3	0.41
CNN + FA^39^	96.1	0.37
CNN + SCA^12^	96.8	0.40
CNN + OBSCA-FS	**98**.**6**	**0**.**43**

The simulation results are presented in the Table 12, where the best accuracy and dropout probability have been reported. The presented findings indicate that the proposed OBSCA-FS approach obtained the best average accuracy and the dropout probability of 0.43, clearly outperforming all traditional and metaheuristics approaches included in the research.

**Figure 7:**
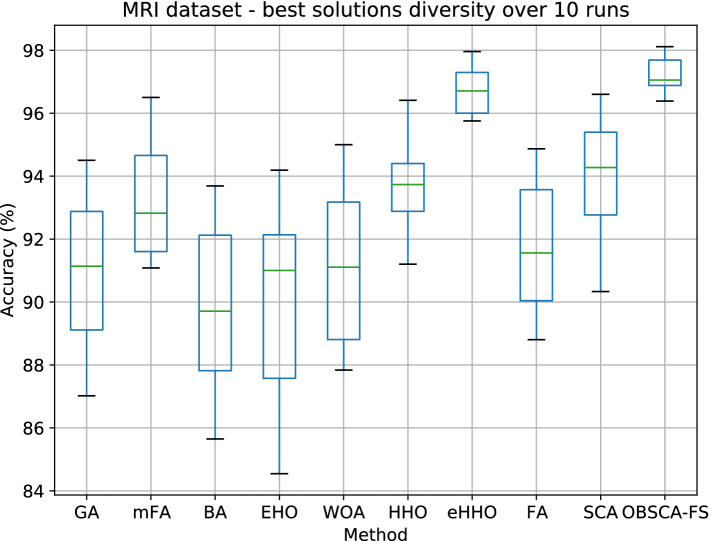
MRI dataset—best solutions diversity over 10 runs results.

**Figure 8:**
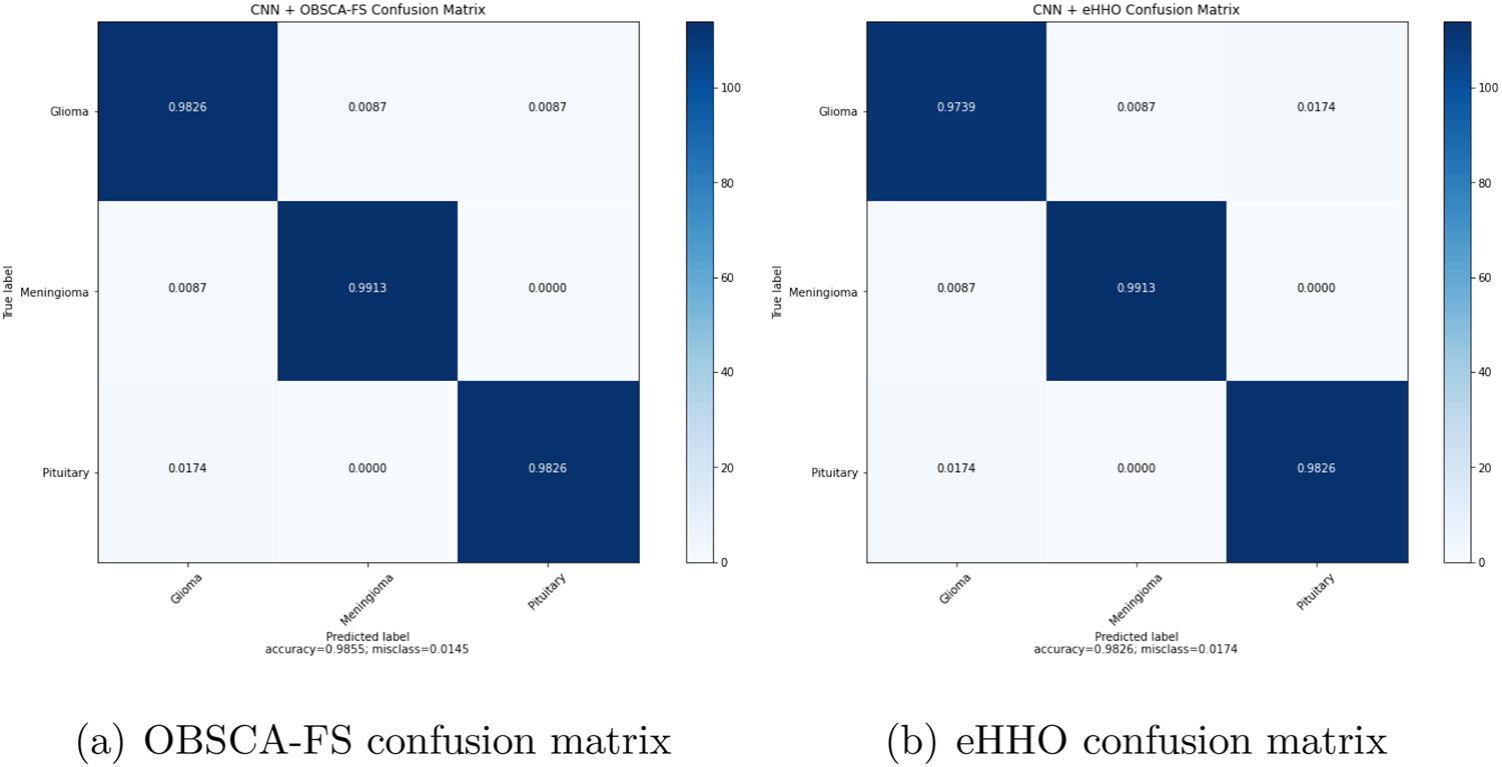
Confusion matrix for OBSCA-FS and eHHO, the two approaches that scored the best results on the MRI dataset.

Figure 7 depicts the box plots for all approaches that were taken in the comparative analysis. It can be noted that the proposed OBSCA-FS approach obtained the best solution diversity. In other words, it has the highest stability over 10 runs, with the smallest standard deviation. Additionally, once more the proposed OBSCA-FS significantly outperformed both SCA and FA methods. Figure 8 shows normalized confusion matrix for two best approaches on the MRI dataset, namely the CNN + OBSCA-FS and CNN + eHHO.

## Conclusion

The research presented in this manuscript proposes a novel automated approach for selecting the regularization dropout parameter *dp* in CNN’s by utilizing the hybridized SCA metaheuristics. The basic SCA is hybridized with the FA’s search equation, moreover, the opposite best solution is generated in each iteration to improve the algorithm’s exploration abilities.

The proposed OBSCA-FS method was first evaluated on 10 recent CEC2019 bound-constrained benchmarks and compared with other state-of-the-art approaches tested with the same experimental data. Reported results, as well as conducted statistical tests, deliver the proof that the proposed method performs significantly better than other approaches. Moreover, it was shown that the OBSCA-FS outscores basic SCA and FA metaheuristics.

Performance of OBSCA-FS was further validated on practical CNN’s application for optimizing dropout probability value, which is very important in preventing overfitting, as one of the most distinguished challenges from the machine and deep learning area. Reported classification accuracy over MNIST, CIFAR-10, Semieon, and USPS datasets clearly shows that the proposed OBSCA-FS has great potential in this domain. The second experiment included the OBSCA-FS practical implementation for MRI classification. The obtained results confirmed the performances of the proposed method as superior.

Due to the great potential of the introduced OBSCA-FS algorithm, in future research, it will be tested on other machine learning challenges and adapted for solving other practical NP-hard problems from the real-world environment. Moreover, the CNN’s regularization will be tackled further by using OBSCA-FS and other similar approaches by fine-tuning other parameters like $\eta$, $\alpha$ and $\lambda$.
